# Participatory Development and Preliminary Psychometric Properties of the User-Friendly Patient Information Material Checklist (UPIM-Check)

**DOI:** 10.3390/ijerph18168773

**Published:** 2021-08-19

**Authors:** Sandra Salm, Judith Mollenhauer, Carolin Hornbach, Natalia Cecon, Antje Dresen, Stefanie Houwaart, Anna Arning, Andrea Göttel, Kathrin Schwickerath, Holger Pfaff, Nadine Scholten, Theresia Krieger

**Affiliations:** 1 Faculty of Human Sciences and Faculty of Medicine, Institute of Medical Sociology, Health Services Research, and Rehabilitation Science (IMVR), University of Cologne, Eupener Str. 129, 50933 Cologne, Germany; judith-m-@t-online.de (J.M.); carolin.hornbach@uk-koeln.de (C.H.); natalia.cecon@uk-koeln.de (N.C.); antje.dresen@uk-koeln.de (A.D.); holger.pfaff@uk-koeln.de (H.P.); nadine.scholten@uk-koeln.de (N.S.); theresia.krieger@uk-koeln.de (T.K.); 2 House of the Cancer Patient Support Associations of Germany (HKSH-BV), Thomas-Mann-Str. 40, 53111 Bonn, Germany; stefanie.houwaart@posteo.de; 3 Cancer Society North Rhine-Westphalia (KG-NRW), Volmerswerther Str. 20, 40221 Düsseldorf, Germany; arning@krebsgesellschaft-nrw.de (A.A.); goettel@krebsgesellschaft-nrw.de (A.G.); schwickerath@krebsgesellschaft-nrw.de (K.S.)

**Keywords:** quality assessment instrument, instrument development, psychometric properties, pilot study, patient information material, patient engagement, service provider engagement

## Abstract

The aims of this study were (1) to design a user-friendly instrument to assess and optimize patient information material (PIM), (2) to develop an English version, and (3) to test its psychometric properties. The instrument was needed to optimize the top-down developed PIM of the psycho-oncological care programme isPO. First, a literature-based PIM checklist was developed by a team of patient representatives, cancer care experts and professional researchers. Next, the checklist’s reliability and validity were analysed by having cancer survivors assess the initial and optimized version of the isPO-leaflet. The *User-friendly Patient Information Material Checklist (UPIM-Check)*, developed participatorily, was found to be effective for evaluating PIM. It uses a traffic light scale, and suggestions for improvement can be given for each criterion. Its reliability appeared to be excellent (α = 0.927). The optimized leaflet was rated significantly better than the initial one. The UPIM-Check is a reliable and valid instrument, which enables end-users (e.g., patients) to assess and optimize the quality of PIM according to scientific criteria and the needs of end-users. A bottom-up approach was essential for developing and validating the UPIM-Check. End-users constantly contributed with their specific knowledge. Thus, their position as co-researchers was significantly strengthened.

## 1. Introduction

### 1.1. Patient Information Material (PIM)

Patient information material (PIM) is written and audio-visual media developed to provide general information on a disease, its early detection, diagnosis and treatment or coping, without presupposing prior medical knowledge [1]. Such information is provided so patients can make health-related decisions [1] and are empowered to communicate with service providers [2]. Therefore, PIM is also of particular importance in intervention studies. Convincing PIM contributes to the acceptance and effective use of interventions [2]. Existing sets of criteria for PIM can guide the development of new PIM. They are similar in terms of structural, content-related and graphic requirements [1,3,4]. Furthermore, it is recommended to involve end-users in the development of PIM to ensure they contain easily readable, understandable and valid information for the end-users [1,3]. Thus, it may be useful to also determine criteria for PIM together with end-users.

With this article, we would like to present how a user-friendly instrument to assess and optimize PIM was designed with end-user engagement. This is exemplified by the participatory optimization process of PIM in the German project isPO (integrated, cross-sectoral Psycho-Oncology).

### 1.2. The Psycho-Oncological Care Programme isPO and Its Project-Specific PIM

**Figure 1:**
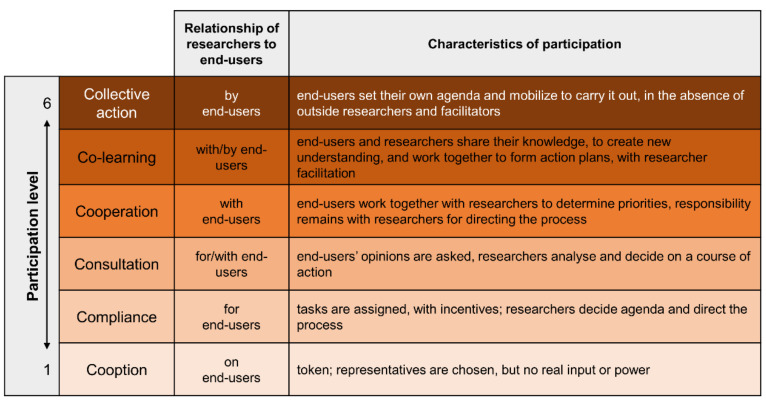
Participation levels, relationship of researchers to end-users, and characteristics of participation adapted from Cornwall (1996, p. 96).

The isPO project is developing, implementing, and evaluating a new, needs-driven psycho-oncological care programme [5,6]. The design process of this complex psycho-oncological intervention proved to follow a top-down approach [7]. Similarly, the project-specific PIM for patient recruitment and study information also was developed with minor end-user participation. Both patients and service providers reported that the PIM for isPO (isPO-PIM) was too extensive, partly redundant and not linguistically appropriate for end-users (newly diagnosed cancer patients). This led to low programme acceptance by patients and especially made patient recruitment challenging. Therefore, the PIM was optimized by a team of cancer patient representatives from the House of the Cancer Patient Support Associations of Germany (HKSH-BV), experts from the Cancer Society North Rhine-Westphalia (KG-NRW) and health researchers of the University of Cologne. The entire optimization process [8] was guided by the participatory health research (PHR) approach according to Cornwall [9], with its six participation levels (Figure 1). Conducting PHR is a process of power-sharing, so participation levels can vary during the course of a project. The aim was to attain the 5th level of co-learning.

The isPO-PIM optimization process gave rise to the need for a PIM assessment instrument following existing quality criteria. For the systematic evaluation of the isPO-PIM and the systematic collection of improvement suggestions at the same time, a PIM assessment and optimization instrument was needed. It was important to develop it with patient engagement, so that it corresponds to the PHR approach. Furthermore, existing PIM quality criteria had to be followed.

### 1.3. PIM Assessment Instruments

Besides the various sets of criteria for the quality of PIM, there are several validated instruments to assess PIM quality [2,10,11,12,13]. According to Clayton [12], PIM assessment instruments can be divided into three types—(1) attribute checklists, (2) readability tests, and (3) rating scales. Several literature reviews on PIM assessment instruments revealed the DISCERN as a commonly used rating instrument [2,4,12] (see below). Due to its specification to PIM on treatment choices and considering certain PIM quality criteria, new instruments have been developed in recent years [2,12,13]. Most of these rating instruments already have been applied in the context of cancer care [14,15,16,17,18,19,20].

In German-speaking countries, DISCERN [11] and the Patient Education Materials Assessment Tool (PEMAT) [13] have been used to assess cancer-related PIM [21,22].

Of these two instruments, only DISCERN was designed to be applied by professionals in health care, health research, and health policy as well as patients. Patients took part in the development and pilot testing of DISCERN, but not in the evaluation of its reliability. Both DISCERN and PEMAT are solely quantitative instruments assessing the quality of PIM with rating scales. Text fields for documenting improvement suggestions are not provided. Regarding the content, DISCERN focuses on the reliability and completeness of information, whereas PEMAT addresses structural and graphical requirements of PIM and assesses their actionability.

### 1.4. Objective of This Study

There was no PIM quality instrument available which could be intuitively applied, especially by PIM end-users. Moreover, existing instruments cannot be directly used for assessment as well as optimization of PIM, e.g., with open text fields for improvement suggestions. More particularly, the usually applied German-speaking PIM instruments do not cover all PIM quality areas. Hence, within the process of optimizing the isPO-PIM, a criteria-based **U**ser-friendly **P**atient **I**nformation **M**aterial **Check**list (UPIM-Check) was developed with perpetual engagement of end-users. It was designed to enable quantitative assessment as well as the optimization of PIM.

In this paper, we report on: (1) the participatory development of the UPIM-Check, (2) the translation process into English, and (3) the psychometric pilot study of the German version.

## 2. Materials and Methods

### 2.1. Ethical Approval

The study was performed according to the Declaration of Helsinki. The ethics committee of the Medical Faculty of the University of Cologne has approved the isPO project and its study design (No. 18-092; date of approval: 15 October 2018). The relevant national and European data protection regulations were considered for data collection. The isPO project study is registered in the German Clinical Trials Register (No. DRKS00015326).

### 2.2. Development of the UPIM-Check (German Version)

**Figure 2:**
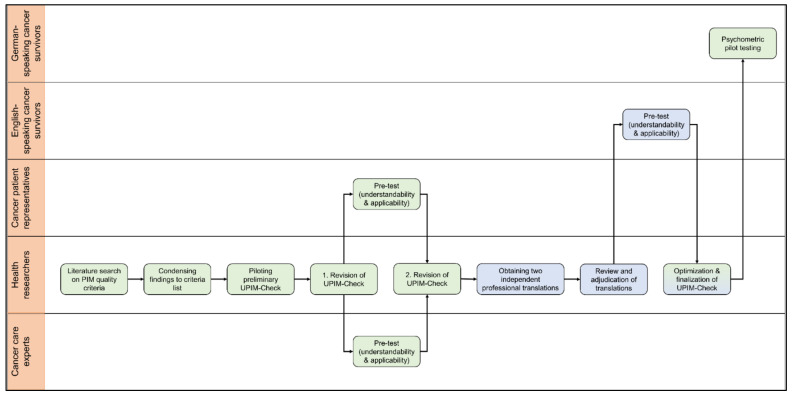
Process of the UPIM-Check development, translation, and psychometric pilot study. The steps are assigned to the group who facilitated and/or conducted this action. Steps in green represent the UPIM-Check German version, steps in blue represent the UPIM-Check English version.

The development of the UPIM-Check took place in a multi-step process, which is shown in Figure 2. It was part of the preparation phase within the isPO-PIM optimization process [8], so the team composition of patient representatives, cancer care experts and researchers was identical.

The design of the new PIM quality instrument was based on the literature on quality criteria and creation manuals for written PIM [1,2,3]. In addition to the written isPO-PIM (leaflets and information folders) there is also an isPO website for patients who are interested in taking advantage of the isPO care programme. So, literature on web-based PIM was also included [4]. The quality criteria that were found were first condensed to a set of 27 criteria by one person from the group of researchers and then grouped into four areas based on the quality indicators of Herm and Linden [3]. This preliminary version of the UPIM-Check was structured into the two sections *Correctness and validity of content* (Does the information appear to be correct and valid?) and *Readability* with the three sub-sections *Readability of content* (Is the content easy to read and the used language appropriate for the end-users?), *Structural readability* (Is the word and sentence complexity appropriate for the end-users?), and *Graphical readability* (Is the graphical design appropriate for the end-users?). For an intuitive assessment of the criteria, a three-point traffic light scale was chosen. The new instrument had to be designed to both assess and optimize PIM, so evaluation and improvement suggestions come from one source to make the instrument efficient. Therefore, an open text field for suggestions for improvement was assigned to each criterion. Three further researchers from the isPO-PIM team piloted the preliminary version of the UPIM-Check and added four items based on the literature mentioned above.

To pre-test the UPIM-Check, it was presented to the other isPO-PIM team members (*n* = 5; two patient representatives and three cancer care experts) to test it on the isPO-PIM. They were asked to critically review it in terms of comprehensibility and applicability. Improvement ideas were inserted on the UPIM-Check form and the UPIM-Check was adapted. In particular, patient representatives suggested linguistic changes to improve clarity and to use language that would empower patients (i.e., motivating them and guiding their actions).

### 2.3. English Translation of the UPIM-Check

To make the UPIM-Check internationally available, an English-language version was generated according to the TRAPD approach (**T**ranslation, **R**eview, **A**djudication, **P**re-test, and **D**ocumentation) [23,24]. The basis was the pre-tested and adapted UPIM-Check German version.

Two professional translators independently translated the UPIM-Check into English (T). A team of four researchers who were active in (psycho-)oncological health services research was formed. Three of them were involved in the development and/or piloting of the preliminary UPIM-Check German version. The fourth person was a native English speaker with eight years of German language experience. Together, they reviewed the two English translations in relation to the German version (R). As the two translations were very similar, linguistic subtleties had to be discussed (e.g., “appropriate” vs. “adequate”). To consent the final English version (A), linguistic consistency within the instrument and an easy language were guiding this step.

The pre-test (P) of the English version had to take place in written form instead of face-to-face interviews, so a special UPIM-Check pre-test form was created. It had an open text field below each criterion so respondents could enter comments on the comprehensibility and relevance of the criterion. In addition, a final text field was available for general comments on the UPIM-Check. To make the UPIM-Check applicable and the comments comparable, the same PIM was made available to all English version pre-test participants. Freely available leaflets on psycho-oncological care in English-speaking countries were researched using an internet search engine. The isPO-PIM team then selected a leaflet published by the national health system of an English-speaking country based on seriousness and independence. The pre-test documents included an invitation letter explaining the aim of the UPIM-Check and how the pre-test would be conducted.

Several methods were used to recruit participants for the pre-test of the English version: The contacts of the HKSH-BV to English-speaking sister organizations were used, and further cancer self-help associations in the UK and USA were identified. In each case, the chairpersons were contacted by email and asked to distribute the pre-test documents to their members. Nine organizations were contacted in up to two contact attempts.

One person each from four organizations provided written feedback on the UPIM-Check. That feedback was incorporated into the final English version of the UPIM-Check. In the pre-test of the English version, it was suggested for some items to formulate them as questions. To be consistent throughout the instrument, the criteria were still formulated as key terms, but questions were added to every item, guiding its assessment (e.g., “*Neutral language*—*Is the PIM presented in an open-minded, not manipulation way?”; “Illustrations*—*Are the pictures and graphics used concise and understandable?”*). Consequently, these questions were also added to the German version to align the both language versions.

### 2.4. Pilot Study on the Psychometric Properties of the UPIM-Check German Version

#### 2.4.1. Pilot Study Design and Participant Recruitment

Since the UPIM-Check was originally developed in German, this version was tested for its psychometric properties first. It was presented to cancer survivors who had no connection to the isPO project or the development of the UPIM-Check. Two versions of the isPO leaflets were assessed—the initial one and the optimized one. To avoid bias because of preconceptions, the study invitation stated only that two leaflets on psycho-oncological care were presented, but not that they were based on an optimization process. Two study groups were defined: (1) participants who evaluated the initial leaflet first and the optimized one second, and (2) participants who evaluated the optimized leaflet first and the initial one second.

Participants for the pilot study were recruited from: (1) the ten associations in the patient organization HKSH-BV, (2) the eight associations in the self-help section of the cancer care expert organization KG-NRW, and (3) Facebook groups organized by and for cancer patients. Up to two contact attempts for each organization were made.

To guarantee their anonymity, interested persons were asked to provide their postal address to the isPO-Trust Centre, which was separate from the research staff in terms of personnel and space.

The recruits were randomly assigned to one of two study groups and given numbers to be used as pseudonyms. Two copies of the UPIM-Check were given to each person, one for each version of the isPO-leaflet.

At the end of the pilot study, all participants received a written report on the results and the UPIM-Check German version for free use.

#### 2.4.2. Materials

The following materials were sent to the pilot study participants by the isPO-Trust Centre to carry out the two assessments: an application guide and two envelopes, each containing a copy of the UPIM-Check and one of the two leaflets. The application guide was a one-page document explaining step-by-step how to conduct the UPIM-Check pilot testing. In this way, the pilot study participants were instructed to open the envelopes and apply the UPIM-Checks in the specified order. The envelopes were numbered according to the study group. For participants in group 1, envelope no. 1 contained the original leaflet and envelope no. 2 contained the optimized leaflet; for study group 2, the numbering was reversed.

After they completed the UPIM-Checks, the participants were asked to return them to the isPO-Trust Centre in an enclosed return envelope.

The UPIM-Check form used for the psychometric pilot study contained some additional elements for study purposes. These included demographics such as age, gender and tumour entity. The time efficiency of the UPIM-Check was also measured. Participants used text fields to enter the times when the UPIM-Check was started and completed. This information was used to calculate the completion time in minutes.

The traffic light scale of the UPIM-Check was operationalised as a three-point Likert scale with the following options: 1 = *very good*; 2 = *sufficient*; 3 = *unsatisfactory*. The UPIM-Check total score and the four criteria areas as subscale scores were calculated as the sum of the corresponding items, with a lower score representing a better rating.

#### 2.4.3. Statistical Analysis

To explore the psychometric properties of the German UPIM-Check, item acceptance, discriminatory power, internal consistency, and construct validity were tested. All analyses were performed to assess both the initial and the optimized isPO-leaflet.

*Item acceptance* was assessed by the completion rate; the higher the rate, the greater the acceptance of the item [25]. For each item, the percentage of pilot study participants who answered it was calculated.

*Item discrimination* is the extent to which the differentiation of subjects within an item corresponds to that within the total score [26]. Thus, the corrected item–scale correlations were computed. This was the correlation between an item and the score of the remaining items [27]. Item–total correlations >0.20 were considered acceptable [28,29].

*Internal consistency* is a measure of the inter-relation of items [30]. Hence, Cronbach’s α [31] was calculated, with values between 0.0 and 1.0. Values >0.70 were considered acceptable, values >0.80 good and values >0.90 excellent [30].

*Construct validity* refers to whether an instrument really measures the construct that it claims to measure [30]. As construct validity cannot be directly measured, its assessment comprises the formulation of hypotheses concerning the relationships of constructs [29,30]. To assess the evidence for the construct validity of the UPIM-Check, the following hypothesis was formulated:

**Hypothesis** **1:** *The UPIM-Check total score correlates highly positively with the four UPIM-Check subscale scores*.

Furthermore, we explored the ability of the UPIM-Check to discriminate between an initial and an optimized PIM, and the time-efficiency of the instrument:

**Hypothesis** **2:** *The UPIM-Check total score on the optimized isPO-leaflet is significantly lower than the total score on the initial leaflet*.

**Hypothesis** **3:** *The UPIM-Check total score correlates positively with the completion time since a lower rating requires more improvement suggestions*.

To test the first and third hypotheses, Spearman’s correlation was calculated one-sided and interpreted according to Taylor [32]: 0.0 to 0.35 = weak correlation; 0.36 to 0.67 = moderate correlation; 0.68 to 1.0 = high correlation. For the second hypothesis, the Wilcoxon test was performed as a non-parametric test for dependent samples.

To test the hypotheses, a significance level of α = 0.05 was assumed and adjusted to α = 0.0125 for hypothesis 1 and to α = 0.025 for hypothesis 3 according to the Bonferroni correction.

Data input, preparation and analysis were performed using IBM SPSS Statistics 27. The data were input manually by the first author, and they were checked by another author, using a dual control principle. For missing values, pairwise deletion was applied.

## 3. Results

### 3.1. The UPIM-Check—Structure and Application

The final UPIM-Check is an instrument for both assessing and optimizing the quality of PIM. It has 31 items, which are divided into four quality areas: *Q1: Correctness and validity of content* (9 items), *Q2: Readability of content* (8 items), *Q3: Structural readability* (4 items), and *Q4: Graphical readability* (10 items).

To map *Q1*, items were developed to assess the information basis of the PIM. The relevance of such information is decisive for the end-users, as well as whether the PIM gives concrete recommendations for action. *Q2* focuses on whether the PIM is designed in such a way that the end-users are specifically addressed and the linguistic design corresponds to the end-users. Delimited from this superordinate level, *Structural Readability* (*Q3*) focuses on features at the word and sentence levels. *Q4* takes up the graphic design of the PIM. This refers to the information provided by illustrations and to the visual design and structuring of the text.

**Figure 3:**
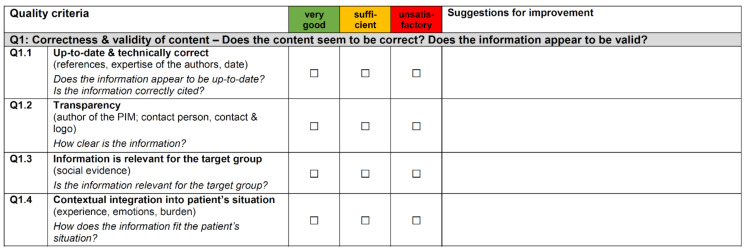
Excerpt from the User-Friendly Patient Information Material Checklist (UPIM-Check) English version. Available at https://www.imvr.de/wp-content/uploads/UPIM-Check_English.pdf, accessed on 18 August 2021.

The items of the UPIM-Check represent concrete criteria and contain questions to help users assessing the criterion. The evaluation itself is quantitative, using a traffic light scale with the levels *very good* (green), *sufficient* (yellow) and *unsatisfactory* (red). For its function as an optimization tool, the UPIM-Check provides an open text field for suggestions for improvement for each criterion. The field can be used if a criterion is rated yellow or red. Figure 3 shows an excerpt from the English version of the UPIM-Check.

On the first page of the UPIM-Check, information about the assessment process itself can be given: (1) name of the PIM, (2) role of the rater (e.g., patient/patient representative, researcher/project staff, care provider), and (3) information on where and how the end-users come into contact with the PIM. Section 2 is intended to support the cooperation of persons with different perspectives in the development, assessment and optimization of PIM, and Section 3 aims to classify the PIM within a communication strategy. That is, if several PIMs with different objectives and different end-users were to be used in a project. The UPIM-Check is freely available at https://www.imvr.de/wp-content/uploads/UPIM-Check_English.pdf, accessed on 18 August 2021.

### 3.2. Preliminary Psychometric Properties of the UPIM-Check German Version

A total of 18 cancer survivors took part in the psychometric pilot study, although one person completed the UPIM-Check only on the optimized leaflet. Pilot study participant characteristics are presented in Table 1.

Item acceptance ranged from 88.2% to 100% for the initial leaflet and from 83.3% to 100% for the optimized leaflet (Table 2). The item with the lowest acceptance was *Q2.8 Use of empowering words*.

The corrected item–total correlations ranged between −0.175 and 0.943 for the initial leaflet and between −0.395 and 0.626 for the optimized leaflet. A total of 26 items for the initial leaflet and 17 items for the optimized leaflet had a corrected item–total correlation of >0.20 (Table 2). Three items were removed from the reliability analysis of the optimized leaflet because their variance was zero. These were *Q2.4 Neutral language*, *Q3.1 Sentence length* and *Q4.7 Font size*. Cronbach’s α was 0.927 for the initial leaflet and 0.655 for the optimized leaflet.

The mean UPIM-Check total score was *M* = 44.08 (*SD* = 10.86) for the initial leaflet and *M* = 38.36 (*SD* = 4.48) for the optimized leaflet. Corresponding information on the subscale scores can be found in Table 2. On average, participants needed 25.18 min (*SD* = 28.09 min) to complete the UPIM-Check for the initial leaflet and 21.18 min (*SD* = 20.27 min) to complete the UPIM-Check for the optimized leaflet.

The scores on the Wilcoxon test were significantly lower for the total score for the optimized leaflet than for the initial leaflet (Z = −2.606; *p* = 0.009). This showed that the UPIM-Check could discriminate PIM versions of different quality.

The Spearman’s correlations of the UPIM-Check total score with the subscale scores for the initial leaflet ranged from *r* = 0.594 to *r* = 0.923. The correlations with the subscales *Q1: Content correctness and validity* and *Q2: Content readability* were significant (*p* < 0.001) (Table 3). Regarding the optimized leaflet, the correlations ranged from *r* = 0.301 to *r* = 0.753, with significant correlations with subscale Q1 (*p* = 0.001) and subscale Q2 (*p* = 0.006). The correlation with subscale *Q4: Graphic Readability* was also significant (*p* = 0.004).

The respective correlations of the UPIM-Check total score with the duration of completion for the two versions of the leaflet showed a significant positive correlation for the initial leaflet (*r* = 0.685; *p* = 0.010) and the optimized leaflet (*r* = 0.606; *p* = 0.014).

The results show that the UPIM-Check is a precise instrument and efficient to use.

## 4. Discussion

**Figure 4:**
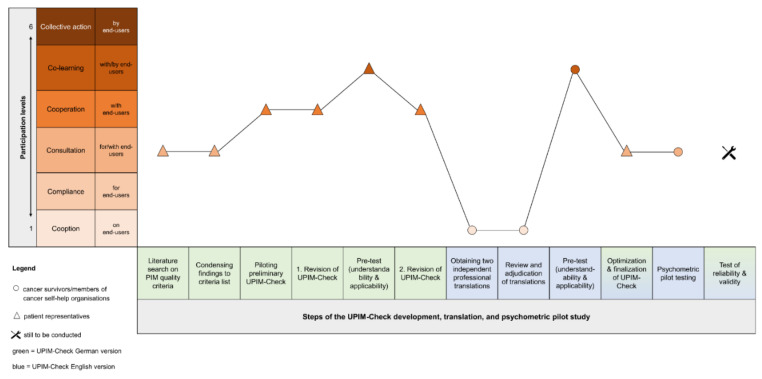
Levels of end-users’ participation (according to Cornwall (1996)) within the steps of the UPIM-Check development and translation process and psychometric pilot study.

This work shows how an end-user-friendly quality instrument for written PIM (UPIM-Check) was developed with ongoing end-user engagement (Figure 4). Besides, it serves as a structured assessment and optimization tool for PIM. The availability of the instrument in German and English widens its usability. Preliminary data on the psychometric properties of the German version were presented first. Validation of the English version is still to be conducted, for which we are looking for interested cooperation partners.

The UPIM-Check was developed to be (1) intuitively usable by all PIM end-users, (2) applicable for both assessment and optimization of PIM and (3) based on scientific criteria for the quality of PIM.

All isPO-PIM team members found their team composition (patient representatives, cancer care experts, and researchers) to be fruitful and enlightening. In particular, the end-users were strengthened in their roles as co-researchers, so that collaboration took place on an equal footing. Moreover, exploring three different perspectives provided a valuable impetus to make the UPIM-Check scientifically and linguistically appropriate, and applicable for end-users. Patient representatives offered the following observations: *‘The checklist covers a lot of aspects, so you get a “close up” of the material’.* There is a *‘reasonable assurance that patients would be able to comprehend the checklist’.*

Nevertheless, the PHR approach also involves some bottlenecks. Krieger et al. [33] found that communication between different groups was very resource-intensive. Knowing this beforehand, we carefully planned and implemented the communication channels between the isPO-PIM team members. It was essential to provide an equal level of information to all team members, as ‘knowledge is power’, and the PHR approach demands power balance [34]. Also, recruiting people from the end-user group during the COVID-19 pandemic was challenging, e.g., in studies with older people and in oncological trials [35,36].

In the psychometric pilot study, an initial and an optimized leaflet for the psycho-oncological care programme isPO were assessed with the UPIM-Check by cancer survivors of various patient organizations representing different tumour entities. Thus, diversity of perspectives was achieved.

While the *internal consistency* of the initial leaflet was excellent, it was just below an acceptable value for the optimized version. However, comparability was limited because only 28 of the 31 items were included in the analysis of the optimized leaflet because some variances were zero. This could lead to an underestimation of reliability [37]. The low dispersion in the evaluation of the optimized leaflet could also indicate a high rater agreement, so PIM of very high quality could be achieved especially for three criteria (*Q2.4 Neutral language*, *Q3.1 Sentence length*, *Q4.7 Font size*).

The values of the item–total correlations as measures of *item discrimination* were acceptable for 26 items (initial leaflet) and 17 items (optimized leaflet), respectively. Four items had a very low item –total correlation of <0.20 for both leaflets. These were: *Q1.1 Up-to-date and technically correct; Q1.9 Further literature/points of contact; Q4.6 Coloured headings and highlighting of key points; Q4.8 Font colour*. If these results are replicated in a representative validation study, items with no acceptable discrimination will have to be removed from the UPIM-Check, as they make no contribution to measure the construct [29].

Regarding *construct validity* (hypothesis 1), it was particularly restrictive that *Q3: Structural readability* was not significantly related to the overall rating. There might be a separate construct that should be addressed with comprehensibility tests [3]. The low acceptance of the empowering language criterion might be related to the average age of the pilot study participants (*M* = 65.28 years) and less familiarity with such formulations (e.g., post-war generation). The empowerment approach is relatively recent, especially in health care [38].

Hypotheses 2 (*discrimination ability*) and 3 (*time-efficiency*) were accepted. This showed that the UPIM-Check could distinguish between an initial and an optimized leaflet. Because of the open text fields, which were completed in the case of low scores, it is plausible that a worse overall score is associated with a longer completion time. Although data were collected by both scale and open text fields, the UPIM-Check proved to be very time-efficient and manageable.

### Strengths and Limitations

The UPIM-Check was developed in an ongoing project (isPO). By pursuing the bottom-up PHR approach, we avoided a research-to-practice gap [34]—in contrast to the top-down development of the initial isPO-PIM. Despite all the constraints (e.g., scarce resources, experts’ scepticism), the participatory procedure was indispensable for optimizing the PIM to create a tailored fit for end-users (cancer patients) and usability in care. The process had to be conducted as quickly as possible to facilitate further programme implementation and enhance patients’ programme acceptance with optimized PIM. Due to this highly practical setting and bottom-up approach on the one hand, and the rarity of similar development processes on the other hand, not every UPIM-Check development step was approached systematically, such as the selection of criteria/items based on a systematic review on PIM quality criteria.

With a sample size of *n* = 18 and two assessed leaflets, the results of this study are not representative. They only give first indications on the psychometric properties of the UPIM-Check German version, so a comprehensive validation still has to be conducted. However, as the UPIM-Check’s observation units are PIM and not people, especially the number of assessed PIMs has to be increased. For example, reliability testing for the DISCERN was conducted by only two raters assessing 31 PIM [17].

The UPIM-Check has been used only in the context of psycho-oncology so far, but its open format implies a high likelihood that it can be applied in a wide variety of care areas. This could generate larger PIM samples for future validation studies like for DISCERN [17] and PEMAT [13]. Also, the full potential of user groups in validating has not yet been realized.

Moreover, construct validity of the UPIM-Check was explored within the instrument, instead of testing the relationships with other PIM assessment instruments (convergent validity) [29,30].

Besides participatory development (Figure 4), the mixed-methods response format consisting of a traffic light scale and open text fields is a unique feature of the UPIM-Check, e.g., in comparison to DISCERN and PEMAT. Knapp et al. [39] conducted PIM optimization based on quantitative user assessments and qualitative user interviews. They achieved a significantly higher understandability and acceptability of the improved PIM, whereas top-down PIM optimization using solely quantitative assessments resulted in minor differences in recruitment rates [40].

Unlike DISCERN and PEMAT, the items of the UPIM-Check do not cover a special area of PIM quality criteria. They consider content-related, structural and graphical requirements, whereas DISCERN focuses on reliability and completeness of information, and PEMAT on understandability and actionability. In contrast to UPIM-Check, DISCERN and PEMAT already have been validated [13,17]. Moreover, the items of DISCERN were notably formulated to assess PIM on treatment choices [2]. Such a specification was avoided in the UPIM-Check. Therefore, it can be applied for various PIMs, e.g., on diagnostics and care programmes.

Dissemination and accessibility of the UPIM-Check are guaranteed on the websites of all developers. The instrument is available for international use, as an English version was created following a recognized translation procedure [23,24].

## 5. Conclusions

The participation of the end-users was essential for the precise definition of the PIM quality criteria and the development of an end-user-friendly PIM quality instrument. The resulting UPIM-Check was rated as valid by experts and user-friendly by patients in both language versions. Through the quantitative and qualitative response format, the UPIM-Check enables both the assessment and optimization of PIM. Since both can be conducted with the same instrument, it is also very time-efficient. This was observed in the psychometric pilot study by the end-user group. However, patients often experience that they are hardly involved beyond the participation level of consultation [9,41,42]. With the PHR approach, end-users engaged in the UPIM-Check’s development and psychometric pilot study (Figure 4), assisting the process with their specific knowledge.

With the UPIM-Check, a PIM quality instrument tool has become universally available, which appeared in a pilot study to be reliable and valid. It had already been used for optimizing and developing PIM [8,43], and it proved to be very end-user-friendly. It is aimed to support all groups involved in the development, optimization or evaluation of PIM, i.e., patients, relatives, patient representatives and professionals in health care, research and policy. The intuitive applicability enables end-users, in particular, to evaluate PIM according to scientific criteria. This empowers them to engage in PHR towards co-learning and collective action [9]. In this way, end-users are strengthened in their role as co-researchers.

## Figures and Tables

**Table 1:** Participant characteristics of the UPIM-Check German version psychometric pilot study.

Characteristic	*M (SD)*	Range
**Age (years)**	65.28 (9.49)	42–79
	***f***	**%**
**Gender**		
female	5	27.8
male	13	72.2
**Tumour entity**		
Bladder	11	61.1
Head and Neck	6	33.3
Other	1	5.6

**Table 2:** Item and score characteristics of the UPIM-Check German version according to the rating of the initial and optimized isPO-leaflet.

Item/Score	Initial Leaflet					Optimized Leaflet				
	*M*	*SD*	Fill-In Rate (%)	Corrected Item-Total Correlation	Cronbach’s α	*M*	*SD*	Fill-In Rate (%)	Corrected Item-Total Correlation	Cronbach’s α
**UPIM-Check total**	44.08	10.86			0.927	38.36	4.48			0.655
**Q1: Correctness and validity of content**	12.63	3.65			0.806	11.76	2.33			0.594
Q1.1 Up-to-date and technically correct	1.24	0.56	100	0.052		1.39	0.70	100	0.047	
Q1.2 Transparency	1.06	0.24	100	0.263		1.47	0.72	94.4	0.381	
Q1.3 Information is relevant for the target group	1.13	0.50	94.1	0.727		1.11	0.32	100	0.376	
Q1.4 Contextual integration into patient’s situation	1.56	0.81	94.1	0.791		1.22	0.43	100	0.626	
Q1.5 Focus	1.35	0.61	100	0.897		1.06	0.24	94.4	0.309	
Q1.6 Adequate presentation	1.65	0.79	100	0.665		1.33	0.59	100	0.626	
Q1.7 Motivation and increase of self-efficacy	1.41	0.62	100	0.736		1.22	0.43	100	0.472	
Q1.8 Recommendation for action	1.53	0.62	100	0.736		1.24	0.56	94.4	−0.174	
Q1.9 Further literature/points of contact	1.59	0.80	100	−0.058		2.06	0.80	100	0.145	
**Q2: Readability of content**	11.42	4.21			0.929	9.47	1.68			0.625
Q2.1 Aim of the PIM and target group is identifiable	1.31	0.60	94.1	0.897		1.06	0.24	94.4	0.309	
Q2.2 Clarity of content	1.44	0.63	94.1	0.943		1.29	0.59	94.4	0.359	
Q2.3 Simple, clear language	1.29	0.59	100	0.897		1.11	0.32	100	0.309	
Q2.4 Neutral language	1.24	0.56	100	0.727		1.06	0.24	100	^a^	
Q2.5 Target group-specific language	1.53	0.74	88.2	0.603		1.39	0.70	100	0.626	
Q2.6 Use of numbers	1.13	0.35	88.2	0.263		1.18	0.39	94.4	0.023	
Q2.7 Language that can be understood without prior medical knowledge	1.75	0.78	94.1	0.795		1.44	0.62	100	0.070	
Q2.8 Use of empowering words	1.60	0.74	88.2	0.895		1.40	0.63	83.3	.292	
**Q3: Structural readability**	5.19	1.76			0.822	4.59	1.12			0.803
Q3.1 Sentence length	1.18	0.39	100	0.068		1.17	0.51	100	^a^	
Q3.2 Sentence difficulty/complexity	1.24	0.44	100	0.264		1.12	0.33	94.4	−0.146	
Q3.3 Word length	1.29	0.59	100	0.374		1.17	0.38	100	−0.146	
Q3.4 Word difficulty	1.56	0.73	94.1	0.248		1.39	0.61	100	0.177	
**Q4: Graphical readability**	13.67	3.54			0.803	12.33	2.50			0.607
Q4.1 Layout/overall visual appearance	1.59	0.71	100	0.226		1.33	0.49	100	0.412	
Q4.2 Eye-catching	2.00	0.73	94.1	0.452		1.59	0.80	94.4	0.208	
Q4.3 Appropriate overall text length	1.35	0.70	100	0.576		1.28	0.58	100	0.316	
Q4.4 Structure and context	1.12	0.33	100	0.263		1.33	0.69	100	0.330	
Q4.5 Illustrations	1.76	0.83	100	0.590		1.69	0.95	88.9	0.425	
Q4.6 Coloured headings and highlighting of key points	1.24	0.44	100	−0.175		1.22	0.43	100	−0.160	
Q4.7 Font size	1.19	0.54	94.1	0.724		1.00	0.00	94.4	^a^	
Q4.8 Font colour	1.18	0.39	100	−0.061		1.06	0.24	94.4	−0.271	
Q4.9 Font type	1.18	0.39	100	0.620		1.11	0.32	100	−0.395	
Q4.10 Corporate design	1.25	0.58	94.1	0.825		1.33	0.59	100	0.286	

Note. ^a^ Item was removed from reliability analysis because it has a variance of zero.

**Table 3:** Spearman’s correlations of the UPIM-Check total score with subscale scores according to the initial and optimized isPO-leaflet.

Subscale Score	UPIM-Check Total Score (Initial Leaflet)		UPIM-Check Total Score (Optimized Leaflet)	
	*r*	*p*	*r*	*p*
Q1: Correctness and validity of content	0.919 *	<0.001	0.753 *	0.001
Q2: Readability of content	0.923 *	<0.001	0.645 *	0.006
Q3: Structural readability	0.637	0.013	0.301	0.148
Q4: Graphical readability	0.594	0.021	0.679 *	0.004

Note. * Significant correlation according to α = 0.0125.

## Data Availability

The data presented in this study are available on reasonable request from the corresponding author. The data are not publicly available due to ethical and legal restrictions (participants of this study did not agree for their data to be shared publicly).
